# Plasma and Red Blood Cell PUFAs in Home Parenteral Nutrition Paediatric Patients—Effects of Lipid Emulsions

**DOI:** 10.3390/nu12123748

**Published:** 2020-12-05

**Authors:** Antonella Lezo, Valentina D’Onofrio, Maria Paola Puccinelli, Teresa Capriati, Antonella De Francesco, Simona Bo, Paola Massarenti, Paolo Gandullia, Marta Marin, Liliana Derevlean, Letizia Baldini, Filomena Longo, Antonella Diamanti

**Affiliations:** 1 Dietetics and Clinical Nutrition Unit, Children’s Hospital Regina Margherita, AOU Città della Salute e della Scienza di Torino, 10126 Torino, Italy; 2 Department of Medical Sciences, Faculty of Medicine, Università degli Studi di Torino, 10126 Torino, Italy; donofriovalentina.vdo@gmail.com (V.D.); simona.bo@unito.it (S.B.); 3 Laboratory of Clinical Biochemistry “Baldi e Riberi”, Metabolic Diseases Unit, AOU Città della Salute e della Scienza di Torino, 10126 Torino, Italy; mpuccinelli@cittadellasalute.to.it (M.P.P.); pmassarenti@cittadellasalute.to.it (P.M.); 20011269@studenti.uniupo.it (L.D.); 4 Artificial Nutrition Division, Ospedale Bambino Gesù, 00165 Roma, Italy; email: teresa.capriati@gmail.com (T.C.); antonella.diamanti@opbg.net (A.D.); 5 Dietetics and Clinical Nutrition Department, AOU Città della Salute e della Scienza, 10126 Torino, Italy; adefrancesco@cittadellasalute.to.it; 6 Pediatric Gastroenterology Department, IRCCS Giannina Gaslini, 16147 Genoa, Italy; paolo.gandullia@gmail.com (P.G.); martamarin@gaslini.org (M.M.); 7 Post-graduate School of Pediatrics, Università degli Studi di Torino, 10126 Torino, Italy; letizia.baldini@unito.it; 8 Pediatric Department, Microcythemia Center, AOU San Luigi Gonzaga, Orbassano, 10043 Torino, Italy; filomena.longo@unito.it

**Keywords:** parenteral nutrition, PUFAs, composite lipid emulsions, fatty acids deficiency

## Abstract

*Background*: Mixed lipid emulsions (LE) containing fish oil present several advantages compared to the sole soybean oil LE, but little is known about the safety of essential fatty acids (EFA) profile in paediatric patients on long-term Parenteral Nutrition (PN). *Aim of the study*: to assess glycerophosfolipid polyunsaturated fatty acids (PUFA) levels on plasma and red blood cell (RBC) membrane of children on long term PN with composite LE containing fish oil (SMOF), and to compare it with a group receiving olive oil LE (Clinoleic^®^) and to the reference range for age, previously determined on a group of healthy children. *Results*: A total of 38 patients were enrolled, median age 5.56 (0.9–21.86) years, 15 receiving Clinoleic^®^, 23 receiving SMOF. Patients on SMOF showed significantly higher levels of eicosapentaenoic acid (EPA) and docosahexaenoic acid (DHA), lower levels of arachidonic acid (ARA) and Mead acid (MEAD)/ARA ratio in plasma and RBC compared with patients on Clinoleic^®^ and with healthy children. Triene:tetraene (T:T) ratio of both groups of patients did not differ from that of healthy children-median plasma (MEAD/ARA: 0.01, interquartile rage (IQR) 0.01, *p* = 0.61 and 0.02, IQR 0.02, *p* = 0.6 in SMOF and Clinoleic^®^ patients, respectively), and was considerably lower than Holman index (>0.21). SMOF patients showed no statistically significant differences in growth parameters compared with Clinoleic^®^ patients. Patients of both groups showed stiffness class F0-F1 of liver stiffness measure (LSM) 5.6 (IQR 0.85) in SMOF patients and 5.3 (IQR 0.90) in Clinoleic^®^ patients, *p* = 0.58), indicating absence of liver fibrosis. *Conclusions*: Fatty acids, measured as concentrations (mg/L), revealed specific PUFA profile of PN patients and could be an accurate method to evaluate nutritional status and eventually to detect essential fatty acid deficiency (EFAD). SMOF patients showed significantly higher EPA, DHA and lower ARA concentrations compared to Clinoleic^®^ patients. Both LEs showed similar hepatic evolution and growth.

## 1. Introduction

Intestinal failure (IF) is a malabsorptive condition characterized by inability of the gut to maintain nutrients and hydration balance, which requires parenteral nutrition (PN) support as a lifesaving therapy [1].

Parenteral nutrition (PN), especially when carried out at home (HPN), is the preferred treatment of intestinal failure (IF) in children. In the last 20 years, the prognosis of IF patients has improved significantly thanks to the global innovation of this therapy and notably to the significant increase of its safety. The development of intestinal failure-associated liver disease (IFALD) is recognized as a limiting factor in long-term management of patients with IF and represents a major indication for intestinal transplantation or combined liver-intestinal transplantation [2,3]. Although the pathogenesis of IFALD is multifactorial, a correlation between the type and dose of lipid emulsion (LE) and the development of cholestatic liver disease has been proposed [4,5]. Specifically, using pure soybean oil LE exceeding 1 g/kg/day has been proved to increase the risk of cholestasis, thus their use is not recommended in long term HPN [6]. Moreover, prolonged restriction of lipid intake in children can lead to essential fatty acid (EFA) deficiency with subsequent adverse effects on growth and neurodevelopment [7]. Pure fish oil lipid emulsions (FO-LE) have shown a dramatic effect on the resolution of cholestasis and an improvement of biochemical measures of hepatobiliary function, compared to pure soybean oil emulsions [6,8,9,10,11,12], but prolonged administration of pure FO-LEs carries the risk of EFA deficiency, although this has not been demonstrated for short term treatment (1 g/kg/day for 1 month) in PN dependent children with IF [13]. Nevertheless, the current ESPGHAN/ESPEN/ESPR/CSPEN paediatric PN guidelines do not recommend long-term administration of pure FO-LEs as the sole source of lipids [7]. Besides, there is insufficient or no evidence of improvement of hepatic fibrosis or extrahepatic outcomes such as growth and cognition with this treatment [14,15,16].

Composite LEs containing fish oil (SMOF-30% soybean oil, 30% medium chain triglicerides-MCTs, 25% olive oil, and 15% fish oil) offer several advantages compared with those containing only soybean oil, including high concentrations of ω-3 PUFAs, DHA and EPA and antioxidant α-tocopherol, reduced ω-6 PUFA content, and a reduced phytosterol load [2,3,7,17].

Evidence from clinical observations indicates that composite LEs with fish oil reduce the risk of cholestasis, oxidative stress and lipid peroxidation; furthermore they provide long chain poliunsatured fatty acids LC-PUFAs (e.g., DHA, which is crucial for neonatal neurodevelopment and vision), have an anti-inflammatory effect due to ω-3 PUFA content, contain a well-balanced ω-6:ω-3 ratio and provide rapidly oxidizable medium-chain fatty acids [8]. There is wide consensus in the scientific community that composite FO-LEs should be considered as a first-line treatment in children on long term PN with cholestasis. If intestinal rehabilitation strategies are unsuccessful, there may be a role for short term use of pure fish oil LEs [7,18]. The use of alternative lipid sources, with or without fish oil, may represent a potential strategy to prevent cholestasis in children with IF, together with promoting oral feeding, which should be administered whenever possible in order to limit the risk of sepsis and small intestinal bacterial overgrowth (SIBO) [7]. Some studies of the previous decade have shown olive oil and fish oil-containing LEs to have nutritional advantages over soybean oil-based LEs and similar safety profile [8,19].

Little is known about the EFA profile safety on long-term administration of composite FO-LEs. A single study [20] on long term administration of SMOF showed a modification of fatty-acid profiles in the red blood cells (RBCs) of children with IF, including remarkably high DHA and EPA levels and significantly low levels of linoleic acid (LA) and arachidonic acid (ARA) at 6 months. These findings were not evidenced by fatty-acid profiles after short time treatment with SMOF [8,18]. In children, more than in adults, EFA profile assessment is crucial, but it is usually performed only for research purposes and expresses PUFA as a relative percentage [21], rather than as absolute plasma concentration. EFAD is usually diagnosed by an elevated triene:tetraene (T:T) ratio. Being a ratio, T:T elevation may reflect either increased oleic acid or Mead acid levels, or a reduced linoleic and linolenic acid level, that may be the case with composite lipid emulsions compared to soybean oil lipid emulsions. Obtaining a fatty acid profile may provide useful information to make a diagnosis of EFAD, in addition to clinical and biochemical signs. Glycerophospholipids, which are hepatic metabolites of ingested or infused lipids, are incorporated in RBC membrane, thus becoming structural lipids. Being not influenced in the short term by the parenteral infusion of LEs, these molecules are an accurate measure of long-term PUFAs status. Recently, a method to quantify PUFAs glycerophospholipids in plasma and erythrocyte membranes by gas chromatography has been validated on healthy children [22]. Here, we apply this method to describe the PUFAs status of children on HPN, and to assess the safety of long-term use of composite LEs.

## 2. Materials and Methods

### 2.1. Aim of the Study

This is a multicenter observational study on PUFA status of 38 patients with IF on long-term HPN. The aim of the study is to assess plasma and erythrocyte PUFAs profile (EPA, DHA, arachidonic acid, MEAD) of paediatric patients on long term use of FO-LE (SMOF^®^) compared to a group treated with olive oil based lipid emulsion (OO-LE) (Clinoleic^®^) and a previously analyzed group of age matched healthy children. Moreover, nutritional, immune and inflammatory status, as well as organ function and liver stiffness, were evaluated in both IF groups.

### 2.2. Inclusion Criteria

We included patients with IF and aged between 0 to 18 years at the time of evaluation and sample collection, who were receiving HPN with the same LE for at least 6 months and at least 3 days/week, with a lipid intake ≥0.5 g/kg/day, followed at three Italian dedicated centers: Regina Margherita Children Hospital (Turin), Pediatric Hospital Bambino Gesù (Rome), Pediatric Hospital Giannina Gaslini (Genoa). The choice of LE was previously and autonomously performed by each Center.

### 2.3. Exclusion Criteria

Patients with end stage liver disease, chronic renal insufficiency, dyslipidemia (type IV), congenital coagulation disorders, recent septic episodes (last month), uncompensated metabolic acidosis or diabetes mellitus, intestinal resections in the last 6 months were excluded.

Healthy Controls (HC): in order to define reference ranges, we utilized plasma and RBC glycerophospholipid PUFA levels of 106 age-matched healthy children, whose samples had been collected and analyzed by the Biochemical Laboratory of Città della Salute e della Scienza of Turin for a previous study [21].

### 2.4. PUFA Profile

For each patient, a quantitative determination of plasma and RBC of the following PUFAs was performed: eicosatrienoic acid or Mead acid (*ω*-9 20:3), arachidonic acid (ARA, *ω*-6 20:4), eicosapentaenoic acid (EPA, *ω*-3 20:5), docosahexaenoic (DHA, *ω*-3 22:6). Later, the following variables were calculated: triene/tetraene ratio (Mead/ARA), and *ω*-6/*ω*-3 ratio (ARA/ (EPA + DHA)). We focused our attention on metabolically critical PUFAs, aiming to optimize a fast, specific, robust and accurate method that might be suitable for routine purposes [23]. In particular, following Koletzko’s method [1,24] at first, polar lipids are selectively extracted from plasma and RBC, then the fatty acids contained in glycerophospholipids are selectively transformed into fatty acid methyl ester derivatives (FAMEs) suitable for gas chromatography (GC) analysis. According to the method developed in our laboratory [23], prepared FAMEs were analyzed through a highly specific gas chromatography-mass spectrometry (GC–MS) approach in Selected Ion Monitoring (SIM) mode and quantitatively determined as mg/L. Specimens analysis was centralized and performed on the same day of the collection. Our method was recently applied to pediatric patients [22].

### 2.5. Data Collection

For each patient a form with anthropometric, clinical and HPN data was filled in the same day of PUFAs sample collection by each center, based on patients’ record files during periodic check-ups. Weight, height, body mass index (BMI) were measured and z-scores for age of each variable were calculated using world Health Organization (WHO) growth charts for children 0–2 years old, Center for Disease Control (CDC) growth charts for children 2–20 years old, on www.peditools.org [25]. Also, HPN characteristics were recorded, including the number of infusions per week, lipid infusions per week, protein intake (g/kg/day; % kcal), glucose (% kcal) and intakes expressed as energy/PN bag or as percentage of basal energy expenditure calculated by Schofield formula. Given the impossibility to quantify the percentage of absorbtion of the ingested PUFAs in patients with IF, we only dealt with PN intake of energy, nutrients and PUFAs.

### 2.6. Biochemical Analysis

For every patient we performed complete blood count, creatinine, glomerular filtration rate (GFR), serum electrolytes, transaminases, glutamylaminotransferase, alkaline phosphatase, total and direct bilirubin, glucose, total and (high density lipoprotein (HDL) cholesterol, triglycerides, low density lipoprotein (LDL) cholesterol (Friedewald formula), coagulation test, total serum proteins, albumin, transferrin, C-reactive protein (CRP), fibrinogen, 25-OH vitamin D, parathyroid hormone (PTH), bone alkaline phosphatase (BAP), vitamin A, E, zinc, copper, selenium—were measured to assess renal and liver function, nutritional, bone, micronutrients status, as well as to detect eventual metabolic complications of HPN. Transient Elastography (Fibroscan, Echosens^®^, Paris, France) was used to determine liver stiffness measurements (LSM), it was performed locally, in centers sharing equal quality criteria (see Supplementary Table S1).

### 2.7. Statistical Analysis

The normal distribution of the data was investigated by Shapiro-Wilk test. Continuous variables were expressed as median, interquartile range (IQR) and minimum-maximum range, while categorical variables as relative frequencies.

Study power analysis defined at least 14 patients/group were necessary for the desired level of significance. Differences between groups were investigated by Mann-Whitney and Kruskal-Wallis test, as appropriate, and considered statistically significant when *p*-value < 0.05. Distribution of plasma and RBC concentrations of PUFA and their correlation were studied by ANOVA F-test, while correlations between numerical variables were established by linear regression analysis. PUFA levels were represented graphically by box plots (1st and 3rd quartile as box limits). Data were analyzed by STATA v12.0 (copyright 1985–2011, StataCorp, College Station, TX, USA).

### 2.8. Ethical Aspects

The study was approved by the local ethical committee, protocol number CS2/1072, on the 9th January 2019. Written informed consent was obtained from all parents or guardians of children.

## 3. Results

A total of 38 patients with IF were enrolled, 15 received OO-LE (Clinoleic^®^) and 23 received FO- LE (SMOF^®^); median age of 5.56 (0.9–21.9) years; 24 (63%) were males.

Patients’ demographic and clinical characteristics, as well as HPN data of the two groups, are reported in Table 1.

**Figure 1:**
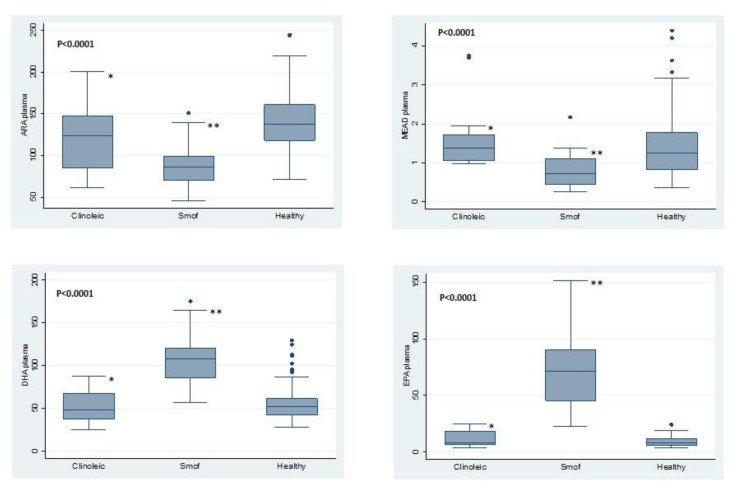
Plasma PUFAs in patients on SMOF or Clinoleic and healthy children: Mead acid (MA); arachidonic acid (ARA); eicosapentaenoic acid (EPA); docosahexaenoic acid (DHA). · *p*-value for the differences between 3 data series are reported in each panel. Under each panel are reported statistically significant differences between 2 groups: SMOF vs. Clinoleic; SMOF vs. healthy children. Differences between 2 groups: ** *p* < 0.01 SMOF vs. Healthy; * *p* < 0.01 SMOF vs. Clinoleic.

**Figure 2:**
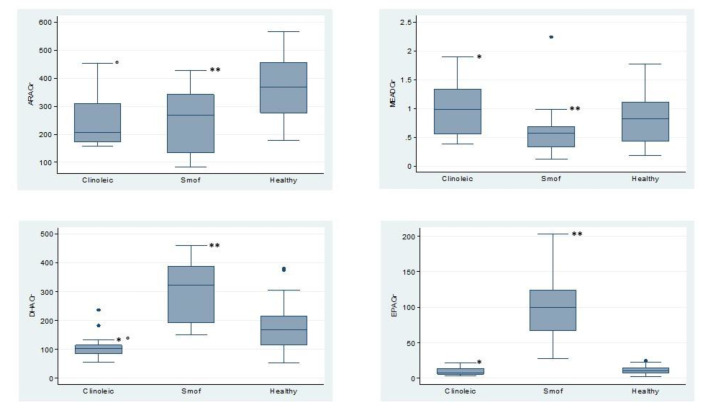
RBC PUFAs in patients on SMOF or Clinoleic and healthy children: Mead acid (MA); arachidonic acid (ARA); eicosapentaenoic acid (EPA); docosahexaenoic acid (DHA). · *p*-value for the differences between 3 data series are reported in each panel. Differences between 2 groups: ** *p* < 0.01 SMOF vs. Healthy; * *p* < 0.01 SMOF vs. Clinoleic; ^°^ *p* < 0.01 Clinoleic vs. Healthy.

No significant difference between the two groups was observed with respect to age, sex and PN characteristics, except for the energy and aminoacidic provision by PN, which was significantly higher in SMOF patients. Also, in the SMOF group more patients presented SBS and congenital mucosal enteropathies, although the overall distribution of IF cause was not statistically significant (Table 1). Glycerophospholipid PUFAs on plasma and RBC of the two groups and of healthy children are represented in Figure 1 and Figure 2.

On one hand, patients receiving composite FO-LE showed significantly higher levels of EPA and DHA on plasma and RBC membranes, on the other and their plasma and RBC levels of ARA and Mead acid were significantly lower, as compared both to patients receiving OO-LE and to HC. (Extensive data in Supplementary Tables S2–S5).

**Figure 3:**
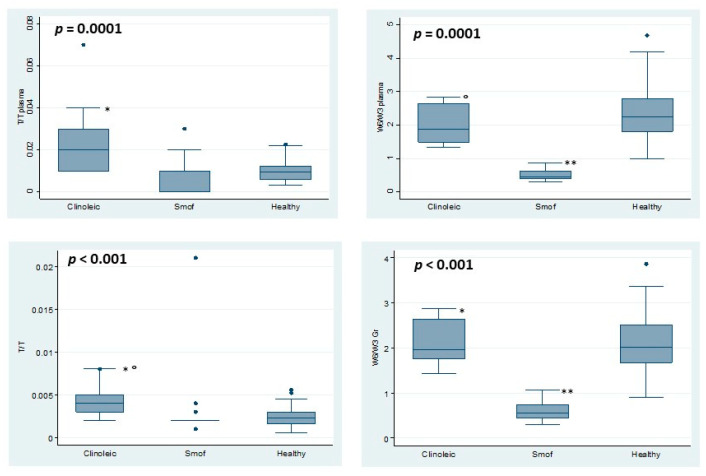
Plasma and RBC MEAD/ARA and ω-6/ω-3 ratio in patients on Smof or Clinoleic and healthy children. MEAD/ARA (Mead/ARA ratio); ω-6/ω-3 (omega3/omeg6 ratio). · *p*-value for the differences between 3 data series are reported in each panel. Differences between 2 groups: ** *p* < 0.01 SMOF vs. Healthy; * *p* < 0.01 SMOF vs. Clinoleic; **^°^** *p* < 0.01 Clinoleic vs. Healthy.

**Figure 4:**
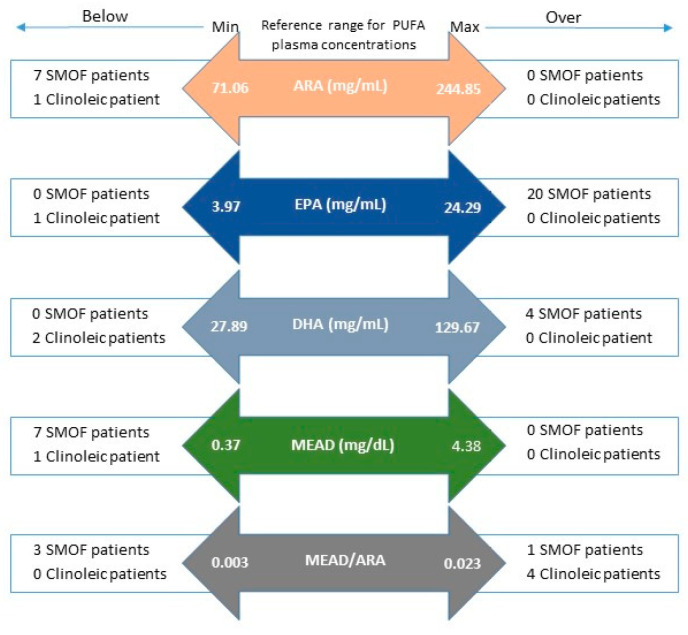
Number of patients on SMOF or Clinoleic having PUFA concentrations below or over the reference range for healthy children. ARA: arachidonic acid; EPA: eicosapentaenoic acid; DHA: docosahexaenoic acid; MEAD: eicosatrienoic acid.

Although FO-LE levels of ARA and Mead/ARA ratio in plasma and erythrocyte were lower, our data did not demonstrated essential fatty acid deficiency (Mead/ARA > 0.21) in these patients—median on plasma Mead/ARA 0.01, IQR 0.01; on RBC 0.002, IQR 0.0; (Figure 3 and Figure 4) (supplementary Tables S2–S5). Patients receiving OO-LE showed a safe Mead/ARA ratio, too (median plasma 0.02, IQR 0.02; RBC 0.004, IQR 0.02). Nonetheless, none of the patients on PN had Mead/ARA > 0.21.

As far as plasma T:T ratio is concerned, both patients on SMOF and Clinoleic did not differ significantly from HC. Nonetheless, statistically significant differences were observed between treatment groups (Figure 3, Supplementary Tables S2–S5). However, dealing with concentrations rather than percentages of fatty acids, we identified some patients having plasma concentrations of PUFAs out of the range used for healthy children. Surprisingly, 4 patients of the Clinoleic group and only 1 patient of the SMOF group had plasma T:T ratio above the reference range (Figure 4).

Omega-6/omega-3 ratio differed significantly between patients and healthy children; specifically, patients on FO-LE had the lowest levels both in plasma and in erythrocyte’s membrane. OO-LE patients had an RBC *ω*-6/*ω*-3 ratio similar to HC, while plasma levels differed significantly (Figure 3, Supplementary Tables S2–S5). Moreover, *ω*-6/*ω*-3 ratio in patients samples differed dramatically from the ratio of their LEs (9:1 in Clinoleic^®^; 2.5:1 in SMOF); as far as FO-LE is concerned, the EPA infused/dosed ratio (mg/L) is lower (0.16, IQR 0.22) than the DHA one (0.6, IQR 0.46)—data not showed.

A stratified analysis of PUFA levels by different age groups (0–2; 2–6; >6 years) was also performed, following the cut-off of age previously suggested [26] (Supplementary Table S6), and showed no statistically significant difference between the two groups of patients, except for plasma DHA in SMOF patients, which tends to decrease from 0–2 to >6 years of age (*p* = 0.0045), (Supplementary Table S6). This phenomenon was not observed in HC.

Furthermore, the correlation between RBC and plasma levels of PUFAs in patients and HC was analysed. Our data demonstrated that plasma concentrations are representative of RBC levels both in patients (*n* = 38) and in controls (*n* = 106): Mead R2 = 0.7206, *p* < 0.001; ARA R2 = 0.0943, *p* < 0.001; EPA R2 = 0.6005, *p* < 0.001; DHA R2 = 0.4604, *p* < 0.001; Mead/ARA R2 = 0.9653, *p* < 0.001; *ω*-6/*ω*-3 R2 = 0.9451, *p* < 0.001 (Supplementary Figure S1).

As far as anthropometric evaluation is concerned, median Z-score of weight, height or BMI for age tended to be lower in FO-LE patients, but differences were not statistically significant if compared to OO-LE group patients (Supplementary Table S7). Nevertheless, 5/23 (21.7%) of patients on FO-LE were malnourished (BMI or weight for age z-score <−2), whilst none of the patients on OO-LE had malnutrition, and height for age z-score was <−2 in 39.1% vs. 13.3%, respectively, although the differences did not reach statistical significance (Table 1). Nonetheless, linear regression analysis showed no correlation among ARA concentrations and any of the anthropometric parameters considered, neither in FO-LE nor in OO-LE patients.

All the biochemical analysis performed, including markers of inflammation, antioxidants, renal and liver function tests, bone, nutritional as well as vitamin and oligomineral status, were similar in both groups of patients (Supplementary Table S8).

Transient Liver elastography was performed in 21/38 patients, 8/23 patients on SMOF—median LSM 5.6 (IQR 0.85) and 12/15 patients on Clinoleic^®^—median LSM 5.3 (IQR 0.90), (*p* = 0.58); patients of both groups showed stiffness class F0-F1, corresponding to no liver fibrosis.

## 4. Discussion

The significant improvement of survival, efficacy and safety of long-term PN in children with IF has to be at least partially attributed to refinements in the composition and delivery of PN, including the use of well-adapted aminoacidic solutions, the avoidance of excess glucose intake, the adoption of cyclical PN infusion, and the development of mixed-oil lipid emulsions. Indeed, IFALD in pediatric patients is not usually caused by the modern PN solutions [27]. First generation soy oil-based LEs are no longer indicated in pediatric IF patients on long term PN [6]. Metabolic benefits of composite third generation LE, containing fish oil, are due to more favorable *ω*-6/*ω*-3 ratio (2.5:1), vitamin E provision (200 mg/100 mL vs. 32 mg/100 mL respectively in SMOF^®^ and Clinoleic^®^) (Table 2), reduced amount of phytosterols and anti-inflammatory and immunomodulatory effects conferred by PUFA *ω*-3 [8].

Two studies on adult patients receiving HPN had consistent results regarding clinical safety and efficacy, demonstrating that composite LE containing olive oil and soya oil were well-tolerated and maintained a normal EFA status, without affecting liver function [29,30]. Another study shown that OO-LEs have a better impact on liver function than soybean oil based LEs in HPN adult patients [31].

Being configured as safety studies, previous studies on adult and pediatric patients had a maximum of 4 weeks of duration [32]. Our study compares PUFAs profile of patients on SMOF^®^ or Clinoleic^®^ for at least 6 months.

Overall, our data suggest the safety profile of composite LEs for long term parenteral use, as shown by the MEAD/ARA ratio. Even if the ratio was far from Holman index value 0.21 (Supplementary Tables S2–S5), none of our patients receiving SMOF^®^ developed EFAD. However, they showed a peculiar PUFA profile when compared to the Clinoleic^®^ group and healthy controls. Considering this, Holman index could not be the best criterion to monitor these patients, as LE may artificially influence individual *ω*-3 and *ω*-6 FAs and the T:T ratio. Consequently, the method used to measure fatty acids and the reference ranges established need to be taken into account, when interpreting these parameters [33].

Furthermore, also biochemical features of EFAD such as elevated liver enzymes, hyperlipidemia and altered platelet aggregation can be a consequence of LE administration [34]. Here, by quantifying fatty acids as concentrations, we highlighted a specific PUFA profile of PN patients, which is consistent with other studies [33,35]. Considering this, we believe that defining specific PUFA range for patients receiving long-term LE by applying our method could provide a more accurate tool to early detect metabolic alterations and, eventually, onset of PN complication.

None of our patients receiving SMOF^®^, which was chosen in high risk patients for its beneficial role on preventing inflammation and cholestasis, developed EFAD. The biologically active *ω*-3 FA molecules (EPA and DHA) contained in SMOF^®^, have been shown to suppress the *ω*-6 cascade, determining a reduction of the relative PUFA *ω*-6 metabolite, arachidonic acid (ARA) [36,37]. Our data are consistent with preliminary data by Goulet et al. in paediatric patients, showing higher DHA and EPA levels and significantly lower levels of linoleic acid and arachidonic acid, after 6–38 months of HPN with SMOF^®^ compared with controls receiving SO-LE [8]. These data were not evidenced in short term treatment [38].

Not only does the use of SMOF assure a safe triene:tetraene ratio, but also it has a non-significant effect on growth, although ARA levels were significantly lower in plasma and RBC of these patients, as compared either to those of the Clinoleic^®^ group or to HC (Figure 1 and Figure 2), consistently with data by Goulet et al. [8]. A recent study showed adequate somatic growth in a large cohort of children with IF treated with FO-LE (Omegaven^®^). However, dealing with data from 2000–2007, the study was unable to evaluate the effects of newer composite IV lipid emulsions [39].

Our study was not powered to investigate all the potential causes of IFALD so we are not able to sustain the exact role of the LE, but these data may be helpful to clinicians. As attended, EPA and DHA concentrations in plasma and RBC of our patients receiving FO-LE were significantly higher if compared with OO-LE patients (Figure 1 and Figure 2). According to a pathophysiological model of non alcoholic steatohepatitis (NASH), impaired hepatic fatty acid desaturation and unbalanced *ω*-6 to *ω*-3 ratio plays a role in the pathogenesis of NASH. Restoration of hepatic *ω*-3 content by an exogenous *ω*-3 enriched diet, significantly reduced intracellular lipid accumulation and inflammatory injury in hepatocytes [40]. Linoleic and alfa linolenic acid provision is similar in the Clinoleic^®^ and SMOF^®^ groups, whilst EPA and DHA provided only by SMOF^®^ might exert additional protective effect on liver [33].

Last but not least, we propose a new method for routine clinical use. Indeed, even if fatty acid composition of erythrocyte membranes is an accurate index of PUFA status [24], as it accurately reflects both oral and intravenous intakes, it is prone to peroxidation artefacts and needs a high complexity laboratory procedure. Conversely, our method to determine plasma concentration of PUFAs has revealed to be simple, less expensive and well correlated with RBC concentrations (Figure 4) [23], reflecting the bioactive fraction of phospholipid PUFAs [41].

In summary, our study demonstrates a peculiar PUFA profile in patients receiving long-term LE; this profile depends on the composite LE administered and is associated with no significant alteration in liver function, biochemical tests and growth parameter. Strengths of our study include its multicenter structure, the long-term follow-up of patients receiving two composite LEs and the innovation of the method, which is applied for clinical purpose for the first time, as far as we known. Weakness include non-quantification of oral intakes and the non-randomization of the LEs, which was independently chosen by the clinicians based on the patients’ needs and could have had an impact on the results.

## 5. Conclusions

Composite lipid emulsions containing fish oil alter PUFA profile in long term HPN patients when compared to healthy children or patients receiving olive oil LE. For that reason, we suggest that determination of fatty acid profile should be part of the complex management of patients on long term PN, to ensure accurate monitoring of long-term PN outcomes. Although no EFA deficiency has been detected in both SMOF^®^ or Clinoleic^®^ patients according to Holman index, some patients of both groups showed a T:T ratio over the upper reference range for healthy children. FA composition of the LE, including the downstream metabolites of PUFA (AA, DHA, and EPA) as well as clinical status must be taken into account while evaluating the risk of EFAD, although the pathogenesis is multifactorial. On the basis of our results, both lipid emulsions assure adequate liver function and growth. High EPA and DHA concentrations provided by SMOF may be of clinical significance. Further studies are needed to confirm our observations and possibly demonstrate our suggestions.

## Figures and Tables

**Table 1:** Patients and HPN characteristics and patients’ age in both lipid groups. Data expressed as median and interquartile range (IQR).

Patients and HPN Characteristics	Clinoleic^®^ *n* = 15 Mediana (IQR)	SMOF^®^ *n* = 23 Mediana (IQR)	*p*-Value
Patients’ age (years)	8.4 (1.6–18.6)	3.3 (0.9–16.9)	0.097
Causes of IF—*n* (%) Short Bowel Syndrome (SBS) Motility disorders (CIPO) Congenital enteropathies Other	9 (60%) 3 (20%) 1 (7%) 2 (13%)	16 (70%) 1 (4%) 4 (17%) 2 (9%)	0.670
PN duration (months)	22.2 (9.8–202)	21.1 (6.9–104)	0.362
PN bags/week	7 (3–7)	7 (5–7)	0.115
Nr lipid infusions/week	6 (3–7)	6 (5–7)	0.643
Lipid intake (g/kg/day)	1.03 (0.5–1.7)	1.3 (0.5–2.5)	0.064
Lipid intake (% kcal)	24.5 (20.1–36.3)	21.5 (6.7–40.3)	0.066
Glucose intake (% kcal)	65.1 (46.0–71.0)	65.2 (48.9–86.1)	0.347
Aminoacid intake (g/kg/day)	1.05 (0.2–1.8)	1.3 (0.7–3.2)	0.002
PN energy provision (Kcal/kg/day)	40.99 (12.0–62.7)	54.4 (23.9–82.8)	0.002
PN energy (% BEE) Energy intake (% BEE)	100 (30–100)	100 (74–118)	0.057
BMI/WFL Z-score <−2	0 (0%)	5/23 (21.7%)	0.046
Weight for age Z-score <−2	3/15 (20%)	13/23 (56.5%)	0.221
Height for age Z-score <−2	2/15 (13.3%)	9/23 (39.1%)	0.050

PN: parenteral nutrition; HPN: home parenteral nutrition; BEE: basal energy expenditure; IF: intestinal failure; BMI: body mass index; CIPO: chronic intestinal pseudoobstruction.

**Table 2:** Lipid emulsion composition; EPA: docosaesaenoic acid; EPA: eicosapentaenoic acid; MCT: medium chain triglycerides. Modified from [28].

Product Name	Clinoleic^®^	Smof^®^ Lipid
Lipid source	20% soybean oil, 80% olive oil	30% soybean oil, 30% MCT, 25% olive oil, 15% fish oil
Soybean oil (g/L)	40	60
MCT (g/L)	0	60
Olive oil (g/L)	160	50
Fish oil (g/L)	0	30
Lipidic composition		
Linoleic acid (%; g/L)	18.5; 18	21.4; 58
α-Linolenic acid (%; g/L)	2; 2	2.5; 6
Arachidonic acid (g/L)	0,6	1
EPA (%; g/L)	0; 0	3; 6
DHA (%; g/L)	0; 0	2; 1
*ω*-6: *ω*-3 ratio	9:1	2.5:1
α-Tocopherol (mg/L)	32	200
Phytosterols (mg/L)	327 ± 8	47.6

## Supplementary Materials

The following are available online at https://www.mdpi.com/2072-6643/12/12/3748/s1, Table S1: Quality requirements/criteria of Transient Elastography (Fibroscan, Echosens^®^, Paris, France); Table S2: PUFAs concentrations in plasma and erythrocyte membranes in HPN patients treated with SMOF^®^ or Clinoleic^®^ and in healthy children; Table S3: PUFAs concentrations on plasma and erythrocyte membranes in HPN patients treated with SMOF^®^ or Clinoleic^®^; Table S4: PUFAs concentrations on plasma and erythrocyte membranes in HPN Patients treated with SMOF^®^ and in healthy children; Table S5: PUFAs concentrations on plasma and erythrocyte membrane profile in HPN Patients treated with Clinoleic^®^ and in healthy children; Table S6: PUFAs concentrations in plasma and red blood cell membranes of HPN patients treated with SMOF^®^ and Clinoleic^®^ divided by age groups; Table S7: Anthropometrics values; Table S8: Laboratory tests: biochemical indices of inflammation, antioxidants, renal and liver function, bone, nutritional as well as vitamin and oligomineral status in patients treated with SMOF^®^ or Clinoleic^®^; Figure S1: Correlation between plasma and RBC PUFAs concentrations.
