# Supplementary material for: Plasma and Red Blood Cell PUFAs in Home Parenteral Nutrition Paediatric Patients—Effects of Lipid Emulsions
Source: Nutrients. 2020 Dec 5;12(12):3748. doi: 10.3390/nu12123748 (PMC7762095; doi:10.3390/nu12123748)
Supplement: Supplementary file 1 [file nutrients-12-03748-s001.zip › Table 1.docx]

**Table S1.** Quality requirements/criteria of Transient Elastography (Fibroscan) ( Echosens ®, Paris, France).

| **Key Performance Indexes** | **Description** |
| --- | --- |
| Technical features | M Probe 3,5 MHz, external ultrasound transducer 7 mm , estimation depth 35-65 mm  Position: suitable intercostals space on the anterior axillary line |
| Stiffness definiton | Propagation velocity (kPa) |
| Measurement Adequacy/appropriateness | 10 valid measurements |
| Measurement validation | Valid measurement if inter-detection IQR <30% |
| Operator’s requirements | Same expert operator |
| Liver Stiffness Measurements (LSM) | <7 kPa no fibrosis; 7-9.5 kPa intermediate fibrosis; >13 kPa advanced/evolutive fibrosis |
