# Supplementary material for: Plasma and Red Blood Cell PUFAs in Home Parenteral Nutrition Paediatric Patients—Effects of Lipid Emulsions
Source: Nutrients. 2020 Dec 5;12(12):3748. doi: 10.3390/nu12123748 (PMC7762095; doi:10.3390/nu12123748)
Supplement: Supplementary file 1 [file nutrients-12-03748-s001.zip › Table 2.docx]

**Table S2.** PUFAs concentrations in plasma and erythrocyte membranes in HPN patients treated with Smof or Clinoleic and in healthy children.

| **PUFA Concentrations in Plasma and Erythrocyte Membranes** | | | | | | | |
| --- | --- | --- | --- | --- | --- | --- | --- |
|  | **SMOF Patients**  **Nr. 23** | | **Clinoleic Patients**  **Nr. 15** | | **Healthy Children**  **Nr. 106** | |  |
| PLASMA | median | IQR | median | IQR | median | IQR | *p-*Value |
| MEAD mg/L | 0.72 | 0.66 | 1.37 | 0.66 | 1.26 | 0.8 | 0.0002 |
| ARA mg/L | 86.2 | 29.56 | 123.47 | 62.51 | 137.3 | 50.4 | 0.0001 |
| EPA mg/L | 71.62 | 45.04 | 8.07 | 12.1 | 8.07 | 60.5 | 0.0001 |
| DHA mg/L | 108.22 | 34.92 | 48.58 | 29.36 | 52.55 | 55 | 0.0001 |
| MEAD/ARA | 0.01 | 0.01 | 0.02 | 0.02 | 0.009 | 0.01 | 0.0001 |
| ω6/ω3 | 0.46 | 0.22 | 1.88 | 1.14 | 2.25 | 1.3 | 0.0001 |
| ERYTHROCYTE |  |  |  |  |  |  |  |
| MEAD mg/L | 0.57 | 0.35 | 0.98 | 0.77 | 0.81 | 0.67 | 0.004 |
| ARA mg/L | 267.24 | 207.24 | 206.55 | 134.2 | 367.17 | 178.6 | 0.0002 |
| EPA mg/L | 100.19 | 56.76 | 7.58 | 7.72 | 11.03 | 6.5 | 0.0001 |
| DHA mg/L | 322.93 | 194.21 | 103.75 | 29.69 | 167.17 | 100.3 | 0.0001 |
| MEAD/ARA | 0.002 | 0 | 0.004 | 0.002 | 0.002 | 0.001 | 0.0002 |
| ω6/ω3 | 0.56 | 0.3 | 1.97 | 0.88 | 2.02 | 0.8 | 0.0001 |

ARA: arachidonic acid; EPA: eicosapentaenoic acid; DHA: docosahexaenoic acid; MEAD: mead acid.
