# Supplementary material for: Plasma and Red Blood Cell PUFAs in Home Parenteral Nutrition Paediatric Patients—Effects of Lipid Emulsions
Source: Nutrients. 2020 Dec 5;12(12):3748. doi: 10.3390/nu12123748 (PMC7762095; doi:10.3390/nu12123748)
Supplement: Supplementary file 1 [file nutrients-12-03748-s001.zip › Table 3.docx]

**Table S3.** PUFAs concentrations on plasma and erythrocyte membranes in HPN patients treated with Smof or Clinoleic.

| **PUFAs Concentrations in Plasma and Erythrocyte Membranes** | | | | | | | | | |
| --- | --- | --- | --- | --- | --- | --- | --- | --- | --- |
|  | **SMOF Patients** | | | | **Clinoleic Patients** | | | |  |
| PLASMA | Min | Median | Max | IQR | Min | Median | Max | IQR | *p-*Value |
| MEAD mg/L | 0.27 | 0.72 | 2.17 | 0.66 | 0.97 | 1.37 | 3.74 | 0.66 | 0.0003 |
| ARA mg/L | 45.29 | 86.2 | 151.2 | 29.56 | 61.28 | 123.47 | 200.62 | 62.51 | 0.013 |
| EPA mg/L | 22.7 | 71.62 | 151.07 | 45.04 | 3.66 | 8.07 | 24.75 | 12.1 | 0 |
| DHA mg/L | 56.89 | 108.22 | 175.34 | 34.92 | 25.49 | 48.58 | 88.05 | 29.36 | 0 |
| MEAD/ARA | 0 | 0.01 | 0.03 | 0.01 | 0.01 | 0.02 | 0.07 | 0.02 | 0.0002 |
| ω6/ω3 | 0.29 | 0.46 | 0.87 | 0.22 | 1.34 | 1.88 | 2.83 | 1.14 | 0 |
| ERYTHROCYTE |  |  |  |  |  |  |  |  |  |
| MEAD mg/L | 0.12 | 0.57 | 2.24 | 0.35 | 0.38 | 0.98 | 1.89 | 0.77 | 0.009 |
| ARA mg/L | 82 | 267.24 | 426.45 | 207.24 | 156.44 | 206.55 | 453.83 | 134.2 | 0.86 |
| EPA mg/L | 27.79 | 100.19 | 203.43 | 56.76 | 3.7 | 7.58 | 22.15 | 7.72 | 0 |
| DHA mg/L | 150.57 | 322.93 | 458.96 | 194.21 | 55.64 | 103.75 | 235.98 | 29.69 | 0 |
| MEAD/ARA | 0.001 | 0.002 | 0.021 | 0 | 0.002 | 0.004 | 0.008 | 0.002 | 0.0001 |
| ω6/ω3 | 0.31 | 0.56 | 1.07 | 0.3 | 1.43 | 1.97 | 2.87 | 0.88 | 0 |

ARA: arachidonic acid; EPA: eicosapentaenoic acid; DHA: docosahexaenoic acid; MEAD: mead acid.
