# Supplementary material for: Plasma and Red Blood Cell PUFAs in Home Parenteral Nutrition Paediatric Patients—Effects of Lipid Emulsions
Source: Nutrients. 2020 Dec 5;12(12):3748. doi: 10.3390/nu12123748 (PMC7762095; doi:10.3390/nu12123748)
Supplement: Supplementary file 1 [file nutrients-12-03748-s001.zip › Table 4.docx]

**Table S4.** PUFAs concentrations on plasma and erythrocyte membranes in HPN Patients treated with SMOF and in healthy children.

| **PUFAs Concentrations on Plasma and Erythrocyte Membranes** | | | | | | | |
| --- | --- | --- | --- | --- | --- | --- | --- |
|  | **SMOFlipid Patients** | | | **Healthy Children** | | |  |
| PLASMA | median | range | IQR | median | range | IQR | *p-Value* |
| MEAD mg/L | 0.72 | 0.27–2.17 | 0.66 | 1.26 | 0.37–4.38 | 0.8 | 0.001 |
| ARA mg/L | 86.2 | 45.9–151.2 | 29.56 | 137.3 | 71.06–244.85 | 50.4 | 0.001 |
| EPA mg/L | 71.62 | 22.7–151.07 | 45.04 | 8.07 | 3.97–24.29 | 60.5 | 0.001 |
| DHA mg/L | 108.22 | 56.89–175.34 | 34.92 | 52.55 | 27.9–129.67 | 55 | 0.001 |
| MEAD/ARA | 0.01 | 0–0.03 | 0.01 | 0.009 | 0.003–0.023 | 0.01 | 0.61 |
| ω6/ω3 | 0.46 | 0.29–0.87 | 0.22 | 2.25 | 0.98–4.68 | 1.3 | 0.001 |
| ERYTHROCYTE |  |  |  |  |  |  |  |
| MEAD mg/L | 0.57 | 0.12–2.24 | 0.35 | 0.81 | 0.18–1.78 | 0.67 | 0.003 |
| ARA mg/L | 267.24 | 82–426 | 207.24 | 367.17 | 177.35–565.2 | 178.6 | 0.001 |
| EPA mg/L | 100.19 | 27.79–203.4 | 56.76 | 11.03 | 2.7–25.07 | 6.5 | 0.001 |
| DHA mg/L | 322.93 | 150.5–458.9 | 194.21 | 167.17 | 53.29–380.2 | 100.3 | 0.001 |
| MEAD/ARA | 0.002 | 0.001–0.021 | 0 | 0.002 | 0.005–0.005 | 0.001 | 0.2 |
| ω6/ω3 | 0.56 | 0.31–1.07 | 0.3 | 2.02 | 0.9–3.86 | 0.8 | 0.001 |

ARA: arachidonic acid; EPA: eicosapentaenoic acid; DHA: docosahexaenoic acid; MEAD: mead acid.
