# Supplementary material for: Plasma and Red Blood Cell PUFAs in Home Parenteral Nutrition Paediatric Patients—Effects of Lipid Emulsions
Source: Nutrients. 2020 Dec 5;12(12):3748. doi: 10.3390/nu12123748 (PMC7762095; doi:10.3390/nu12123748)
Supplement: Supplementary file 1 [file nutrients-12-03748-s001.zip › Table 5.docx]

**Table S5.** PUFAs concentrations on plasma and erythrocyte membrane profile in HPN Patients treated with Clinoleic and in healthy children.

| **PUFAs Concentrations on Plasma and Erythrocyte Membranes** | | | | | | | |
| --- | --- | --- | --- | --- | --- | --- | --- |
|  | **Clinoleic Patients** | | | **Healthy Children** | | |  |
| PLASMA | **median** | **Range** | **IQR** | **median** | **range** | **IQR** | ***p-Value*** |
| MEAD mg/L | 1.37 | 0.97–3.74 | 0.66 | 1.26 | 0.37–4.38 | 0.8 | 0.22 |
| ARA mg/L | 123.47 | 61.28–200.62 | 62.51 | 137.3 | 71.06–244.85 | 50.4 | 0.058 |
| EPA mg/L | 8.07 | 3.66–24.75 | 12.1 | 8.07 | 3.97–24.29 | 60.5 | 0.35 |
| DHA mg/L | 48.58 | 25.49–88.05 | 29.36 | 52.55 | 27.9–129.67 | 55 | 0.42 |
| MEAD/ARA | 0.02 | 0.01–0.07 | 0.02 | 0.009 | 0.003–0.023 | 0.01 | 0.6 |
| ω6/ω3 | 1.88 | 1.34–2.83 | 1.14 | 2.25 | 0.98–4.68 | 1.3 | **0.001** |
| ERYTHROCYTE |  |  |  |  |  |  |  |
| MEAD mg/L | 0.98 | 0.38.1.89 | 0.77 | 0.81 | 0.18–1.78 | 0.67 | 0.297 |
| ARA mg/L | 206.55 | 156.4–453.8 | 134.2 | 367.17 | 177.35–565.2 | 178.6 | **0.0013** |
| EPA mg/L | 7.58 | 3.7–22.1 | 7.72 | 11.03 | 2.7–25.07 | 6.5 | 0.2 |
| DHA mg/L | 103.75 | 55.6–235.9 | 29.69 | 167.17 | 53.29–380.2 | 100.3 | **0.003** |
| MEAD/ARA | 0.004 | 0.002–0.008 | 0.002 | 0.002 | 0.005–0.005 | 0.001 | **0.001** |
| ω6/ω3 | 1.97 | 1.43–2.87 | 0.88 | 2.02 | 0.9–3.86 | 0.8 | 0.8 |

ARA: arachidonic acid; EPA: eicosapentaenoic acid; DHA: docosahexaenoic acid; MEAD: mead acid.
