# Supplementary material for: Plasma and Red Blood Cell PUFAs in Home Parenteral Nutrition Paediatric Patients—Effects of Lipid Emulsions
Source: Nutrients. 2020 Dec 5;12(12):3748. doi: 10.3390/nu12123748 (PMC7762095; doi:10.3390/nu12123748)
Supplement: Supplementary file 1 [file nutrients-12-03748-s001.zip › Table 6 .docx]

**Table S6.** PUFAs concentrations in plasma and red blood cell membranes of HPN patients treated with SMOF and Clinoleic divided by age groups.

| **PUFAs Concentrations by Age Groups in SMOF and Clinoleic Patients** | | | | | | | | |
| --- | --- | --- | --- | --- | --- | --- | --- | --- |
|  | **< 2 years old Pts** | | - 1. **Years old** | | **>6 years old** | |  | |
| **PLASMA** | **SMOF**  **Nr. 6**  **Median (IQR)** | **Clinoleic**  **Nr. 2**  **Median (IQR)** | **SMOF**  **Nr. 9**  **Median (IQR)** | **Clinoleic**  **Nr. 4**  **Median (IQR)** | **SMOF**  **Nr. 8**  **Median (IQR)** | **Clinoleic**  **Nr. 9**  **Median (IQR)** | ***p**** | ***p*^°^** |
| **MEAD mg/L** | 0.49 (0.12) | 1.125 (0.19) | 0.95 (0.67) | 1.38 (0.19) | 0.92 (0.78) | 1,155  (0,97) | 0.5569 | 0.3566 |
| **ARA mg/L** | 98.11 (28.88) | 84.45 (5.18) | 83.9 (26.55) | 126.58 (48.71) | 81.39 (41.08) | 276,95  (179,84) | 0.7312 | 0.4509 |
| **EPA mg/L** | 72.46 (18.26) | 6.35 (1.77) | 63.96 (63.74) | 7.89 (2.49) | 68.34 (31.05) | 12,61  (11,93) | 0.6674 | 0.2532 |
| **DHA mg/L** | 121.08 (12.26) | 47.94 (19.59) | 110.05 (22.15) | 58.03 (36.34) | 86.17 (19.28) | 109,65  (78,21) | **0.0446** | 0.7357 |
| **MEAD/ARA** | 0.00 (0.001) | 0.01 (0.00) | 0.01 (0.00) | 0.03 (0.02) | 0.01 (0.01) | 0,0035  (0,0025) | 0.1167 | 0.1372 |
| **ω6/ω3** | 0.49 (0.18) | 1.61 (0.54) | 0.46 (0.40) | 1.59 (0.68) | 0.475 (0.28) | 2,06  (0,895) | 0.9527 | 0.2315 |
| **ERYTHROCYTE** |  |  |  |  |  |  |  |  |
| **MEAD mg/L** | 0.42 (0.31) | 0.68 (0.00) | 0.57 (0.55) | 0.90 (0.73) | 0.59 (0.17) | 1.16 (0.97) | 0.9827 | 0.6623 |
| **ARA mg/L** | 252.93 (173.15) | 195.97 (0.00) | 269.66 (191.96) | 189.88 (52.2) | 244.73 (212.12) | 276.95 (179.85) | 0.7966 | 0.4089 |
| **EPA mg/L** | 113.94 (19.38) | 6.93 (0.00) | 91.815 (40.35) | 7.07 (2.89) | 95.40 (57.35) | 12.61 (11.93) | 0.1726 | 0.5877 |
| **DHA mg/L** | 322.93 (118.16) | 114.88 (0.00) | 330.73 (194.14) | 95.31 (22.74) | 307.3 (210.29) | 109.655 (78.21) | 0.5004 | 0.4459 |
| **MEAD/ARA** | 0.002 (0.00) | 0.003 (0.00) | 0.002 (0.00) | 0.01 (0.00) | 0.00 (0.00) | 0.00 (0.00) | 0.2215 | 0.6036 |
| **ω6/ω3** | 0.53 (0.30) | 1.61 (0.00) | 0.56 (0.30) | 2.12 (0.81) | 0.59 (0.30) | 2.06 (0.90) | 0.9640 | 0.5639 |

ARA: arachidonic acid; EPA: eicosapentaenoic acid; DHA: docosahexaenoic acid; MEAD: mead acid. *p value inter-group differences SMOF patients. ^°^ p value intergroup differences Clinoleic patients
