# Supplementary material for: Plasma and Red Blood Cell PUFAs in Home Parenteral Nutrition Paediatric Patients—Effects of Lipid Emulsions
Source: Nutrients. 2020 Dec 5;12(12):3748. doi: 10.3390/nu12123748 (PMC7762095; doi:10.3390/nu12123748)
Supplement: Supplementary file 1 [file nutrients-12-03748-s001.zip › Table 7.docx]

**Table S7.** Anthropometrics values.

| Anthropometrics values | Smoflipid Patients  Median (IQR) | Clinoleic Patients  Median (IQR) | *p*-Value |
| --- | --- | --- | --- |
| Weight z-score | −1,95 (2.03) | −0,58 (2.39) | 0.2 |
| Height z−score | −1.58 (2.44) | −1.06 (1.99) | 0.28 |
| (Body Mass Index) BMI z−score | −0.96 (1.98) | 0.52 (2.25) | 0.2 |
