# Supplementary material for: Plasma and Red Blood Cell PUFAs in Home Parenteral Nutrition Paediatric Patients—Effects of Lipid Emulsions
Source: Nutrients. 2020 Dec 5;12(12):3748. doi: 10.3390/nu12123748 (PMC7762095; doi:10.3390/nu12123748)
Supplement: Supplementary file 1 [file nutrients-12-03748-s001.zip › Table 8.docx]

| **Biochemical Analysis** | **Smoflipid Patients**  **Median (IQR)** | **Clinoleic Patients**  **Median (IQR)** | ***p*-Value** |
| --- | --- | --- | --- |
| Total protein (g/dL) | 6.8 (1.7) | 6.7 (0.7) | 0.4 |
| Albumin (g/dL) | 4.3 (1.7) | 4.2 (0.4) | 0.2 |
| Cholinesterasis (UI/ml) | 7042 (2639) | 6469 (2779.5) | 0.4 |
| Lymphocytes (10̂ ̂6/L) | 2.3 (2.25) | 1.96 (1.43) | 0.6 |
| Haemoglobin (g/dL) | 11.2 (2.1) | 11.5 (1.9) | 0.9 |
| Total Cholesterol (mg/dL) | 118 (38) | 99 (25) | 0.7 |
| Triglycerides (mg/dL) | 59 (61.5) | 81 (50) | 0.15 |
| HDL (mg/dL) | 29 (17) | 32 (8) | 0.9 |
| LDL (mg/dL) | 77 (36) | 54.2 (44.5) | 0.2 |
| Glycemia (mg/dL) | 84 (15) | 82 (8) | 0.15 |
| Antioxidants |  |  |  |
| P- Vitamine E (mg/mL) | 10 (6.9) | 9.2 (6.1) | 0.9 |
| p-Vitamine A (mg/mL) | 0.32 (23.76) | 0.37 (0.26) | 0.25 |
| Liver function |  |  |  |
| AST (UI/L) | 44.5 (25) | 39.5 (18) | 0.16 |
| ALT (UI/L) | 30.5 (39) | 28.5 (28) | 0.06 |
| GGT (UI/L) | 20 (42) | 19 (31) | 0.57 |
| Total Bilirubin (mg/dL) | 0.4 (0.2) | 0.4 (0.4) | 0.9 |
| Direct Bilirubin (mg/dL) | 0.2 (0.16) | 0.2 (0.2) | 0.4 |
| ALP (UI/L) | 289.5 (165) | 232.5 (152) | 0.06 |
| INR | 1.25 (12.77) | 1.19 (0.07) | 0.27 |
| Oligoelements |  |  |  |
| Selenium (mg/L) | 60.75 (19) | 62.7 (20.45) | 0.5 |
| Zinc (mg/L) | 0.76 (65.15) | 0.835 (0.155) | 0.3 |
| Copper (mg/L) | 0.9 (0.86) | 1.18(0.68) | 0.46 |
| Cromium (mg/L) | 1.13 (0.77) | 1.03 (0.46) | 0.7 |
| Renal Function |  |  |  |
| Creatinine (mg/dL) | 0.35 (0.23) | 0.32 (0.35) | 0.52 |
| e-GFR (ml/min/1.73mq) | 134.5 (51) | 123.5 (52) | 0.87 |
| Bone status |  |  |  |
| 25OH Vitamina D (ng/mL) | 25.4 (17.6) | 18.9 (9.2) | 0.2 |
| p-Calcium (mg/dL) | 2.25 (6.65) | 2.34 (0.14) | 0.3 |
| p-Phosphorus (mg/dL) | 1.69 (2.69) | 1.45 (0.25) | 0.9 |
| Parathyroid Hormone (pg/dL) | 37 (44.9) | 34.5 (53.5) | 0.7 |
| Bone ALP (UI/L) | 54.2 (47.85) | 44.8 (17.9) | 0.07 |
| Inflammatory status |  |  |  |
| C-Reactive Proteine (mg/L) | 2.1 (0-51) | 0.35 (0-20) | 0.6 |
| Fibrinogen (mg/mL) | 294 (138-471) | 292.5 (196–437) | 0.7 |
| Leucocytes (10 6/L) | 6620 (1260-16240) | \| 6255 (3630–11230) \| \| --- \| | 0.07 |

**Table S8.** Laboratory tests: biochemical indices of inflammation. antioxidants. renal and liver function. bone. nutritional as well as vitamin and oligomineral status in patients treated with SMOF or Clinoleic.

Glomerular filtration rate (GFR). AST: apartateaminotranspherasis. ALT: alanineaminotranspherasis. GGT: glutamylaminotransferase. ALP: alkaline phosphatase. ^°^ *p* < 0.01.
