# Supplementary figures and images for: Plasma and Red Blood Cell PUFAs in Home Parenteral Nutrition Paediatric Patients—Effects of Lipid Emulsions
Source: Nutrients. 2020 Dec 5;12(12):3748. doi: 10.3390/nu12123748 (PMC7762095; doi:10.3390/nu12123748)

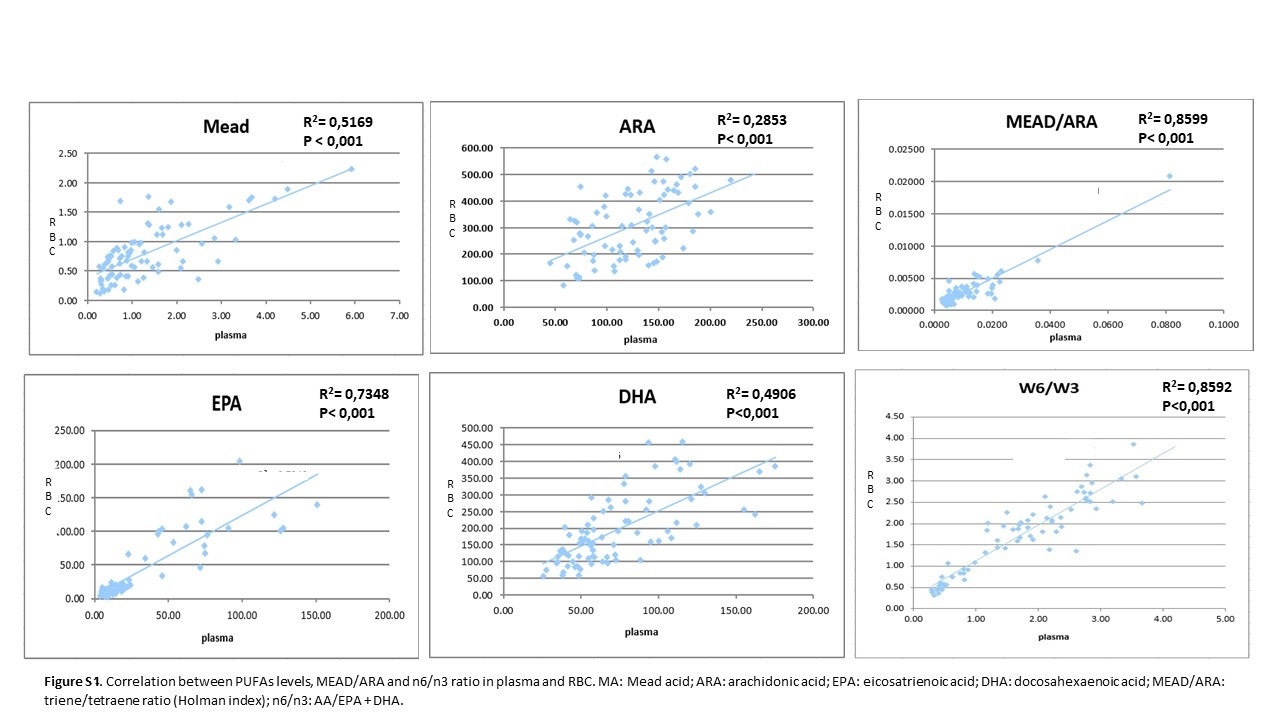

Supplement: Supplementary file 1 [file nutrients-12-03748-s001.zip › Figure S1.jpg]
